# The local immune response during *Echinococcus granulosus* growth in a quantitative hepatic experimental model

**DOI:** 10.1038/s41598-019-56098-3

**Published:** 2019-12-23

**Authors:** Zhide Li, Chuanshan Zhang, Liang Li, Xiaojuan Bi, Liang Li, Shuting Yang, Ning Zhang, Hui Wang, Ning Yang, Abuduaini Abulizi, Abudusalamu Aini, Renyong Lin, Dominique A. Vuitton, Hao Wen

**Affiliations:** 1 State Key Laboratory of Pathogenesis, Prevention, Treatment of High Incidence Diseases in Central Asia, the First Affiliated Hospital of Xinjiang Medical University, Urumqi, Xinjiang China; 2 Department of Hepatic Hydatid and Hepatobiliary Surgery, Digestive and Vascular Surgery Centre, the First Affiliated Hospital of Xinjiang Medical University, Urumqi, Xinjiang China; 3 Xinjiang Key Laboratory of Echinococcosis, and WHO-Collaborating Center on Prevention and Care Management of Echinococcosis, Clinical Research Institute, the First Affiliated Hospital of Xinjiang Medical University, Urumqi, Xinjiang China; 4 Basic Medical College, Xinjiang Medical University, Urumqi, Xinjiang China; 5 Chronic Disease Laboratory, Institutes for Life Sciences and School of Medicine, South China University of Technology, Guangzhou, China; 6 French National Reference Center for Echinococcosis, Department of Parasitology, University Hospital, Besançon, France; 7 University Bourgogne Franche-Comté (EA 3181), Besançon, France

**Keywords:** Inflammatory diseases, Parasitic infection

## Abstract

The local immune mechanisms responsible for the establishment and development of *Echinococcus granulosus sensu stricto* infection in the liver, have been little explored. We developed a suitable experimental model that mimics naturally infected livers using portal injection of protoscoleces. Opposite to *Echinococcus multilocularis* infection which is dose-dependent, fully mature hydatid cysts can be established in the liver whatever the injection dose; although most of the infection sites were seen at the establishment phase as inflammatory granulomas associated with fibrosis, they never matured into cysts. At the establishment phase, a strong immune response was composed of T and B cells, with T1-type, T2-type cells and cytokines and IL-10-secreting CD8^+^ T cells in the liver. At the established phase, results suggested a local production of antibodies by B cells, and an involvement of NK and NKT cells. Infection outcome and local immune response in the liver, were different in the mouse models of *Echinococcus granulosus sensu stricto* and *Echinococcus multilocularis* respectively; however, only early specificities at the microenvironment level might explain the major differences found between the lesions induced by the two species. Our quantitative experimental model appears fully appropriate to further study this microenvironment and its relationship with each cestode species.

## Introduction

Cystic echinococcosis (CE), a severe zoonosis caused by *Echinococcus granulosus sensu lato* (*E. granulosus s.l*.) larvae, is threatening human’s health and social development worldwide^1,2^. CE presents in humans as a cyst, most frequently in the liver^3^. The metacestode is composed of the ‘hydatid’, a cystic structure composed, from inside to outside, of the hydatid fluid (HF), the germinal layer (GL) that produces the protoscoleces (PSCs), the laminated layer (LL), of parasitic origin, and the adventitial layer (AL) produced by the host’s immune reaction. The treatments of CE consist of surgical resection, percutaneous drainage with injection of protoscolex-killing compounds (protoscolecides), and the anti-parasite drug, albendazole^4,5^. Host’s immune system interact with *E. granulosus s.l*. through immune cells and cytokines which contribute to immediate or delayed healing or to the continuous growth of the cyst when infection has been successfully established^6^. The WHO classification of CE cysts in the liver highly reflects the outcome of such a metacestode-host interaction by defining three types of cysts: active (CE1 and CE2), transitional (CE3 a and b), and degenerating/inactive (CE4 and CE5). However, the mechanisms responsible for either self-healing (inactivation) or maintenance of a chronic infection have not been fully elucidated yet. From various studies performed in CE patients and in experimental models, it is commonly accepted that CE cyst formation is mainly mediated by impairment of dendritic cells (DC) differentiation and maturation, and by the differentiation of specialized regulatory T cells and related cytokines such as Interleukin-10 (IL-10) and transforming growth factor beta (TGF-β)^7^. In addition, a feature of *E. granulosus s.l*. infection is its capacity to induce T helper 2 (Th2)-type response, which are beneficial to the survival of the metacestode, rather than T helper 1 (Th1) cytokines, capable of its destruction^8^.

Previously, we developed a suitable experimental mouse model that mimics naturally infected livers by injection of *Echinococcus multilocularis* (*E. multilocularis*) PSCs via the portal vein. This model allowed us to study host’s immune response according to the parasite load. By using this model, we demonstrated that local cellular immunity and fibrogenesis were actually protective and fully able to limit metacestode growth or even to clear it in the liver after low or medium dose-infection, while impairment of cellular immunity was followed by a more rapid and severe course of the disease in high dose-infected mice^9^. Recently, we have adapted this quantitative mouse model to *Echinococcus granulosus sensu stricto* (*E. granulosus s.s*.) to study the effect of innovative treatments of CE^10,11^.

For obvious reasons, the local immune response before and at the early stages of cyst formation in human CE is barely known. Very little information is available on the local immune response and its course in the liver, at the early stages of *E. granulosus* infection in experimental models^12,13^. In addition, marked differences between pathology and clinical course of CE and AE have long been recognized^3,12^ although *E. granulosus* spp. and *E. multilocularis* share most of their DNA sequences^14–16^. However, all immunological studies have shown that the course of T cell differentiation and cytokine secretion were highly similar in both infections, yet no clear immunological explanation has been provided up to now to render these differential pathological and clinical features^12^. Taking benefit from the similarity and quantitative nature of the model of PSC injection in the portal vein for both *E. granulosus* and *E. multilocularis*, we thus investigated the impact of the inoculation of different *E. granulosus s.s*. PSC numbers (corresponding to low, medium and high dose groups, respectively LDG, MDG and HDG) on the development of the cysts in the liver of C57BL/6 mice, a strain of intermediate susceptibility to both species of *Echinococcus*, as is observed in humans, and we assessed the relationship between the observed histological aspects, intensity of infection, and liver fibrosis. To study the relationship between infection following increasing number of PSCs and the host’s immune response, and make the immunological profile observed in this model comparable to that previously described in the experimental model of *E. multilocularis* infection, we measured most of the known cellular and cytokine markers of the immune balance in *Echinococcus* spp. infection and analyzed whether and how these patterns affected the establishment of infection and parasite growth.

## Results

### Establishment of a mouse model by injecting different doses of *E. granulosus s.s*. PSCs via the portal vein

General observation revealed that the hepatic cysts were embedded at the surface of the liver, or in the liver, or were (less frequently) free in the peritoneal cavity. The development of hepatic cysts was observed from the 4^th^ to 24^th^ week after infection; and different courses of infection could be seen in all dose groups (Supplementary Fig. S1). At 2 weeks, tiny white foci less than 1 mm in size were observed on the surface of the liver. Congestive hemorrhagic patches were also observed and an increased number of foci were seen from LDG, MDG to HDG at that stage, and there were no cysts in any of the groups, including HDG (2000 PSCs). At 4 weeks, the foci decreased in number, but the sizes of the foci were larger than those observed at 2 weeks by 1–2 mm. Cysts were seen in one mouse in HDG. At 8 weeks, cysts of 1–4 mm in diameter and 1–10 in number were found mostly at the margin of the liver lobes in MDG and HDG; cysts were observed in only one mouse in LDG (50 PSCs) (Supplementary Table S1). At 12 and 16 weeks, cysts of 1–10 mm in diameter and 1–20 in number were observed in all groups. At 20 and 24 weeks, numerous cysts of 1–15 mm in diameter were found in the liver of all mice whatever the group, and a few cysts in the peritoneal cavity were observed in HDG.

From the 8^th^ week, as the doses of PSCs increased, the mean number of cysts gradually increased, but there was only a weak, albeit significant correlation between the number of cysts/mice and the dose of PSC injected (r = 0.22, R^2^ = 0.05, *p* < 0.05) (Supplementary Table S1). In addition, as the dose of PSCs increased, the mean formation ratio of cysts gradually decreased (Supplementary Table S2). Most cysts were located in the caudate or left lobe of liver.

### Histopathological features and types of lesions in the liver of mice with different *E. granulosus s.s*. PSC inocula

**Figure 1:**
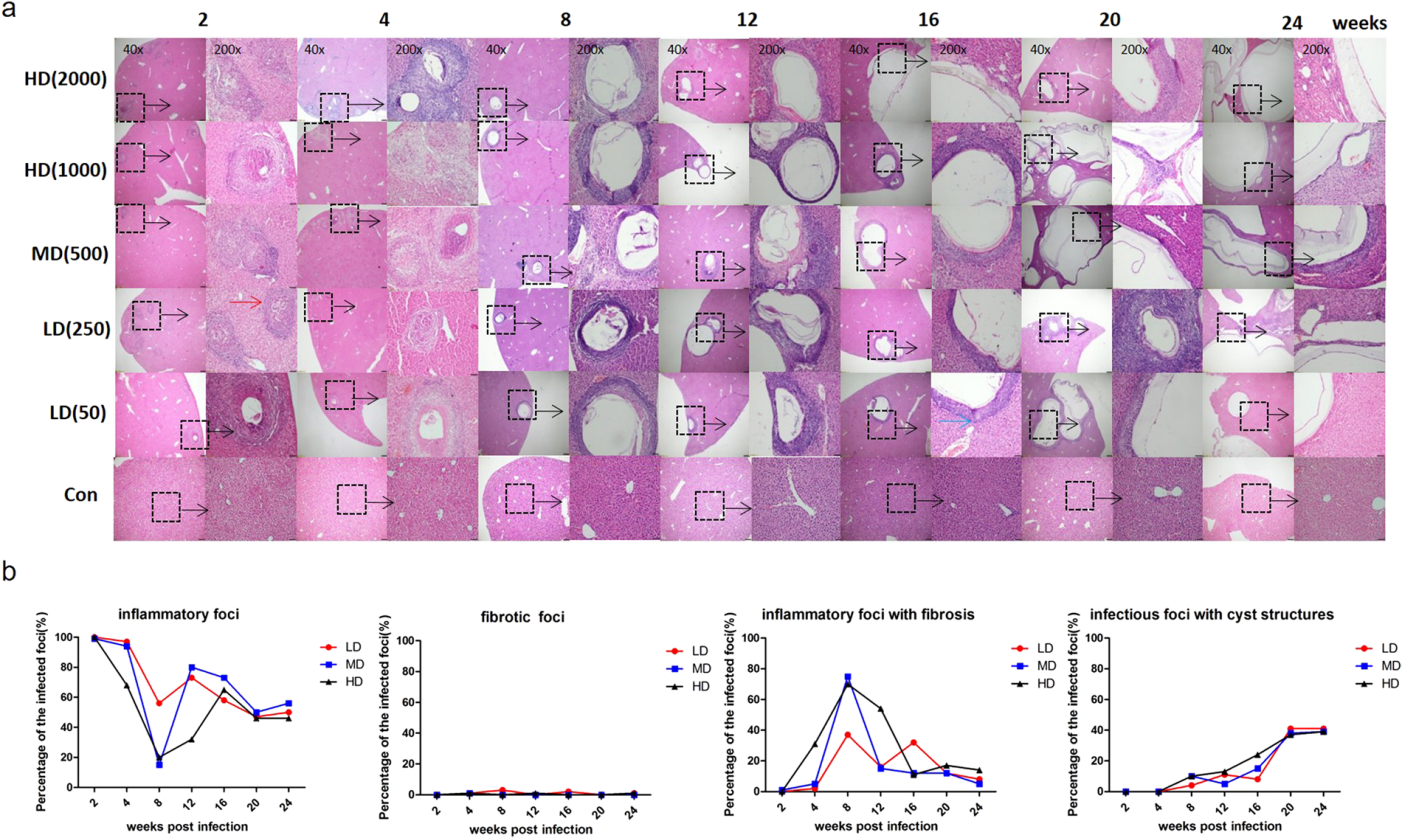
Hepatic histopathological alterations and granulomatous response in mice during the course of infection. (**a**) Histopathological alterations of the liver from mice infected with different PSC inocula during the course of infection. H&E staining of liver sections. The original magnification was at 40×, and the corresponding images on the right were magnified at 200×; bars indicate 200 μm or 50 μm in the 40× or in the 200× magnification images, respectively. Red arrow indicates inflammatory cell zone; Blue arrow indicates fibrosis areas. Dashed line marks the border of granuloma around the parasitic lesion in the infected group. (** b**) Hepatic granulomatous response to *E. granulosus* infection with different doses. Liver histological reaction at each infectious foci was scored as (1) inflammatory foci, parasite-free, except for possible PSC remnants, composed of macrophages, lymphocytes, and other inflammatory cells; (2) fibrotic foci, parasite-free, with no visible PSCs or cysts, only composed of fibrosis; (3) inflammatory foci with fibrosis, parasite-free, with no visible PSCs or cysts, only composed of granulomatous inflammation combined with liver fibrosis; (4) infectious foci, cystic structure composed of the germinal layer and laminated layer, surrounded with macrophages, lymphocytes, fibroblasts, myofibroblasts, as well as fibrosis (adventitial layer). All sizes of infectious foci were calculated via microscope examination of the liver including 4–6 mice per group. PSCs: protoscoleces. LD: 50 or 250 PSCs; MD: 500 PSCs; HD: 1000 or 2000 PSCs. Data are shown as mean ± standard error of the mean (SEM), *p < 0.05, **p < 0.01 and ***p < 0.001.

At 2 weeks, lymphocytes infiltrated the *E. granulosus s.s*. inoculum in all infected groups, and a few foci were surrounded by fibroblasts; a few PSCs could still be observed in the portal vein. At 4 weeks, the cyst formed in a mouse of the HDG was typical with GL, LL and AL. At 8 weeks, when the cysts began to form in all groups and gradually increased in number, at the periphery of the lesion(s), numerous fibroblasts and inflammatory cells were present and an obvious increase of granulomas (inflammatory cells with fibrosis) was observed. From the 12^th^ to 24^th^ weeks, cysts were the main lesions and consisted of the GL, LL and AL composed of picric acid-Sirius red stained fibrosis and of host immune cells, including lymphocytes, macrophages, and epithelioid cells. Cysts were located at the edge of or inside the liver. Some of the cysts contained necrotic tissue. At that stage, the number and size of cysts in HDG were usually larger than that in MDG and LDG. (Fig. 1a).

As shown in Fig. 1b, inflammatory foci occurred most frequently at 2 and 4 weeks, inflammatory foci with fibrosis at 8 and 12 weeks, and infectious foci with cystic structures at 20 and 24 weeks. In all groups, fibrotic foci without inflammatory cell infiltration were rarely observed. In LDG, inflammatory foci decreased from 2 to 24 weeks (from 99.7% to 44.3%). Inflammatory foci with fibrosis increased from 2 weeks and peaked at 8 weeks (36.4% of all foci), then decreased at 24 weeks. Infectious foci with cystic structures appeared at 8 weeks, then gradually increased, and peaked at 20 weeks (54.9% of all foci). In MDG, the inflammatory foci were at the lowest level at 8 weeks (15.3% of all foci). Inflammatory foci with fibrosis increased and peaked at 8 weeks (74.5% of all infectious foci), then decreased at 24 weeks. Infectious foci with cystic structures also appeared at 8 weeks, increased gradually, and peaked at 20 weeks (56.9% of all infectious foci). In HDG, the course of the various types was similar to that observed in MDG. Inflammatory foci were at their lowest level at 8 weeks (18.4% of all infectious foci); inflammatory foci with fibrosis peaked at 8 weeks (66.9% of all infectious foci), and infectious foci with cystic structures peaked at 24 weeks (53.3% of all infectious foci).

### Lesion size and inflammatory cell infiltration in the liver of mice with different *E. granulosus s.s*. PSC inocula

**Figure 2:**
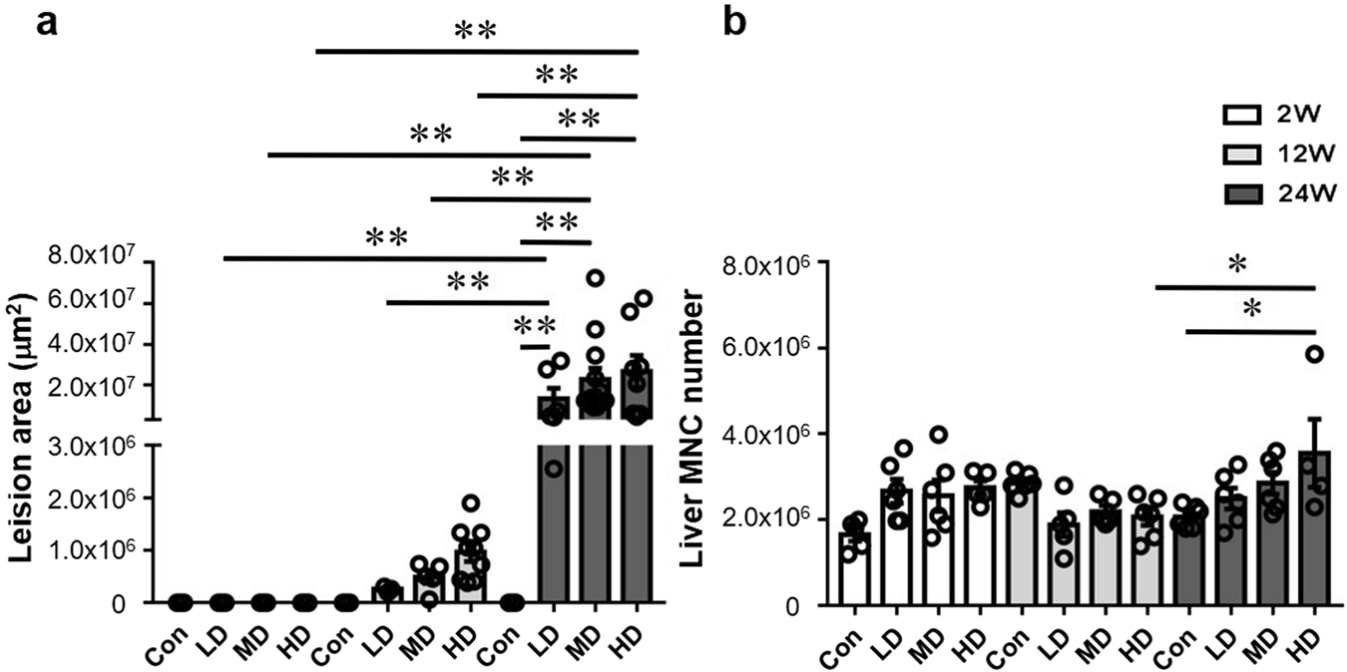
Lesion size and inflammatory infiltration in the liver of mice during the course of infection. (**a**) Liver lesion area determined by microscopic measurement of H&E-stained tissue sections, and expressed as square micrometers (μm^2^). (**b**) Total number of hepatic mononuclear cells (MNCs). LD: 50 PSCs; MD: 500 PSCs; HD: 2000 PSCs. Data are shown as mean ± standard error of the mean (SEM, 4–6 mice per group), *p < 0.05, **p < 0.01 and ***p < 0.001.

No specific identified lesions were observed in the control groups, with sham injection of PSCs, at any post-injection time. At 2 weeks, there was no difference in the size of liver lesions. At 12 weeks, the size of liver lesionswas not significantly different in the three groups. At 24 weeks, the average size of liver lesions in the three groups was significantly higher than at 2 and 12 weeks (Fig. 2a). At 24 weeks, the number of infiltrating mononuclear cells (MNCs) in the liver in HDG was significantly higher than in the control group, and also significantly higher than at 12 weeks post-injection (Fig. 2b).

### Characteristics of hepatic fibrosis in mice with different *E. granulosus s.s*. PSC inocula

At 2 weeks, mild collagen deposition was observed around granulomatous inflammatory lesionsand in the portal spaces, but it was not significantly different in all infected groups. At 12 and 24 weeks, picric acid-Sirius red staining, albeit higher in MDG and HDG than in LDG, was not significantly different in all infected groups. At these stages, collagen deposits were mainly localized around the LL of the cysts, and also in the granulomas without cyst formation (Supplementary Fig. S2a,b).

Cells with α-SMA expression were also present around the cysts and in the granulomas (Supplementary Fig. S2c). At 2 and 24 weeks, the percentage of α-SMA positive cells was significantly higher in HDG and MDG than in the control groups (*p* = 0.0179; *p* = 0.0282; *p* < 0.001; *p* < 0.001). At 24 weeks, the percentage of α-SMA positive cells was higher in HDG than in LDG (*p* = 0.0216). α-SMA positive cells were still present at the late stage of infection, and the percentage of α-SMA positive cells was higher in HDG and MDG at 24 weeks than at 12 weeks (*p* = 0.0024; *p* = 0.0097) (Supplementary Fig. S2d).

### Lymphocyte composition in the liver of mice with different *E. granulosus s.s*. PSC inocula

**Figure 3:**
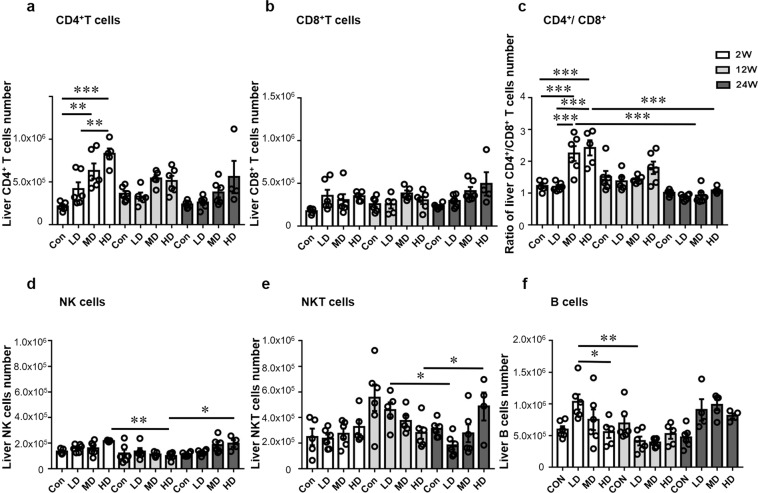
Inflammatory cell subsets in the liver of mice infected during the course of infection. (**a**) Absolute quantification of hepatic CD4 (NK1.1^−^CD3^+^CD4^+^) T cells. (**b**) Absolute quantification of hepatic CD8 (NK1.1^−^CD3^+^CD8^+^) T cells. (**c**) Ratio of CD4^+^ T cells/CD8^+^ T cells in the liver. (**d**) Absolute quantification of hepatic NK (NK1.1^+^CD3^−^) cells. (**e**) Absolute quantification of hepatic NKT cells. (**f**) Absolute quantification of hepatic CD19^+^ B cells. LD: 50 PSCs; MD: 500 PSCs; HD: 2000PSCs. Data are shown as mean ± standard error of the mean (SEM, 4–6 mice per group), *p < 0.05, **p < 0.01 and ***p < 0.001.

The absolute numbers of CD4^+^ T cells were significantly higher in HDG than in LDG at 2 weeks (*p* = 0.003), but the difference was not statistically signifcant at 12 and 24 weeks. The absolute numbers of CD4^+^ T cells were significantly higher in HDG and MDG than in the  control group at 2 weeks (*p* < 0.001; *p* = 0.003). The number of CD8^+^ T cells was not different in the three groups at all three stages (Fig. 3a,b). The CD4^+^/CD8^+^ T cell ratio gradually increased in the liver as the infection dose increased, and it was markedly lower in LDG than in MDG and HDG at 2 weeks (*p* < 0.001; *p* < 0.001). Moreover, the ratio of CD4^+^/CD8^+^ T cells was significantly higher in MDG and HDG at 2 weeks than at 24 weeks (*p* < 0.001; *p* < 0.001) (Fig. 3c).

The number of NK cells was significantly lower in HDG at 12 weeks than at 2 and 24 weeks (*p* = 0.0028; *p* = 0.0457) (Fig. 3d). The number of NKT cells was significantly higher in LDG at 12 weeks than at 24 weeks (*p* = 0.0386), and higher in HDG at 24 weeks than at 12 weeks (*p* = 0.0292) (Fig. 3e). In addition, the absolute number of B cells was higher in LDG at 2 weeks than at 12 weeks (*p* = 0.0011); and it was higher in LDG than in HDG at 2 weeks (*p* = 0.0457) (Fig. 3f).

### Phenotype of memory T cell subsets in the liver of mice with different *E. granulosus s.s*. PSC inocula

**Figure 4:**
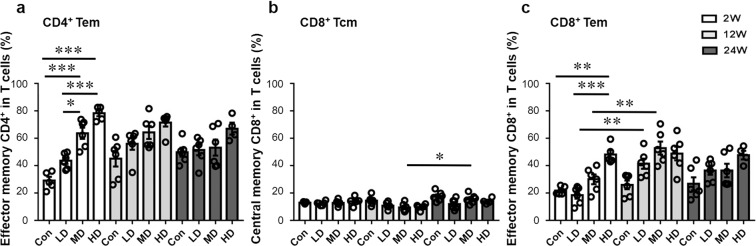
Memory T cell phenotypes in the liver of mice during the course of infection. (**a**) Percentage of effector memory CD4^+^ T cells (Tem, CD44^+^CD62L^−^)/CD4^+^ T cells in the liver. (**b**) Percentage of central memory CD8^+^ T cells (Tcm, CD44^+^CD62L^+^)/CD8^+^ T cells in the liver. (**c**) Percentage of effector memory CD8^+^ T cells (Tem, CD44^+^CD62L^−^)/CD8^+^ T cells in the liver. LD: 50 PSCs; MD: 500 PSCs; HD: 2000 PSCs. Data are shown as mean ± standard error of the mean (SEM, 4–6 mice per group), *p < 0.05, **p < 0.01 and ***p < 0.001.

At 2, 12 and 24 weeks, the percentage of effector memory CD4^+^ T cells (Tem, CD44^+^CD62L^−^) was higher in MDG and HDG than in LDG (*p* < 0.001;*p* = 0.0177), and it was significantly increased in MDG and HDG at 2 weeks, and represented 78.2 ± 4.3% of CD4^+^ T cells in HDG (Fig. 4a and Supplementary Fig. S3).

At 2, 12 and 24 weeks, the percentage of central memory CD8^+^ T cells (Tcm, CD44^+^CD62L^+^) was not significantly different in the three groups. In addition, it was higher in MDG at 24 weeks than at 12 weeks (*p* = 0.0186). The percentage of effector memory CD8^+^ T cells (Tem, CD44^+^CD62L^−^) was higher in HDG than in LDG at 2, 12 and 24 weeks, and it was significantly different at 2 weeks (*p* < 0.001). In LDG, CD8^+^ Tem percentage was higher at 12 than at 2 weeks (*p* = 0.0037). In MDG, CD8^+^ Tem percentage was higher at 12 weeks than at 2 weeks (*p* = 0.0039). CD8^+^ Tem percentage was significantly higher in HDG than in control group, and represented 48.2 ± 6.1% of CD8^+^ T cells at 2 weeks (*p* = 0.0017) (Fig. 4b,c).

### Dynamic changes of T1-type CD4 T cells and CD8 T cells in the liver of mice with different *E. granulosus s.s*. PSC inocula

**Figure 5:**
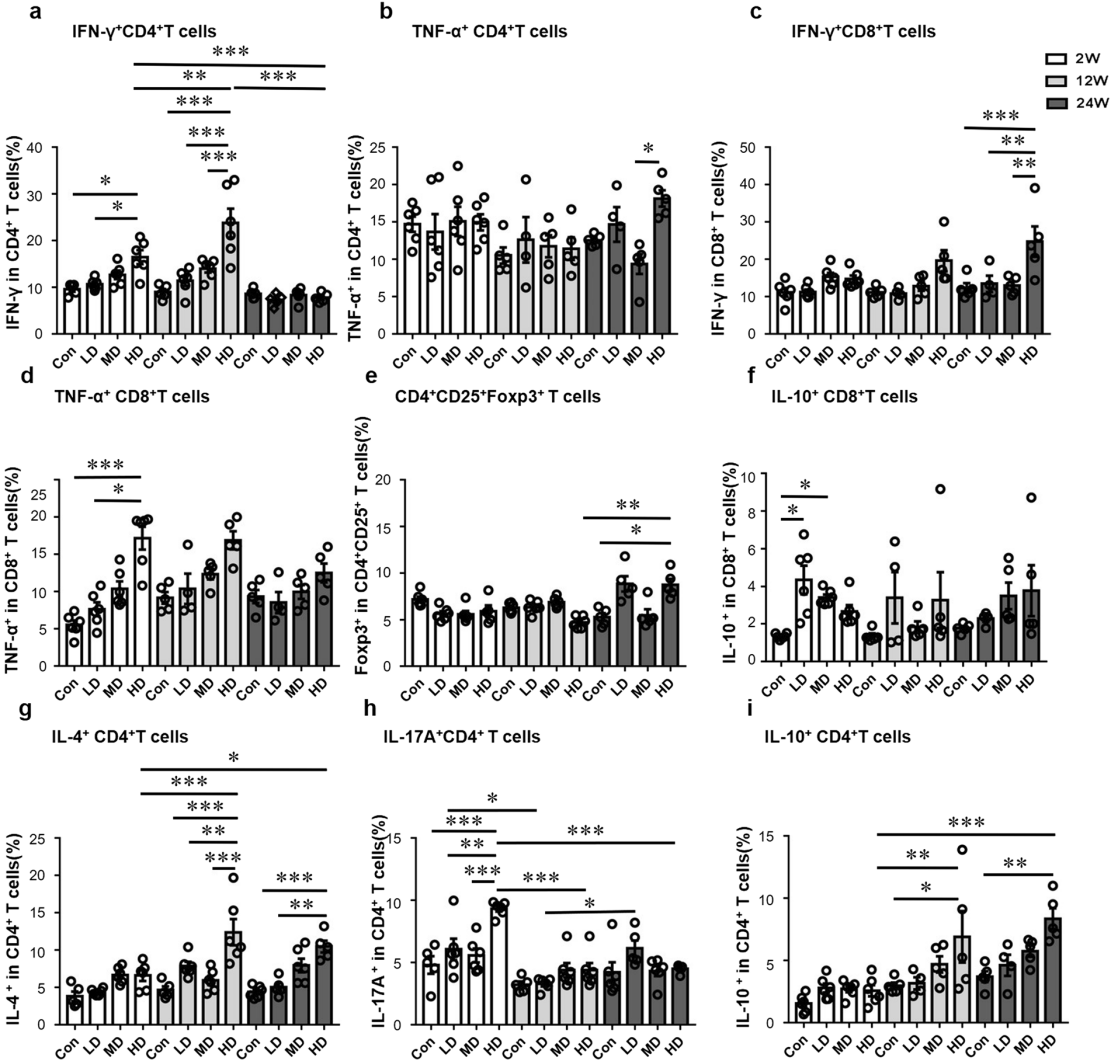
Distribution of T cell subsets in the liver of mice during the course of infection. (**a**) Percentage of CD4^+^IFN-γ^+^ T cells (T1-type)/CD4^+^ T cells in the liver. (**b**) Percentage of CD4^+^TNF-α^+^ T cells (T1-type)/CD4^+^ T cells in the liver. (**c**) Percentage of CD8^+^IFN-γ^+^ T cells (T1-type)/CD8^+^ T cells in the liver. (**d**) Percentage of CD8^+^ TNF-α^+^ T cells (T1-type)/CD8^+^ T cells in the liver. (**e**) Percentage of CD4^+^CD25^+^Foxp3^+^ T cells (Treg-type)/CD4^+^ T cells in the liver. (**f**) Percentage of CD8^+^IL-10^+^ T cells (Treg-type)/CD8^+^ T cells in the liver. (**g**) Percentage of CD4^+^IL-4^+^ T cells (T2-type)/CD4^+^T cells in the liver. (**h**) Percentage of CD4^+^IL-17A^+^ T cells (T17-type)/CD4^+^T cells in the liver. (**i**) Percentage of CD4^+^IL-10^+^ T cells (Treg-type)/CD4^+^T cells in the liver. LD: 50 PSCs; MD: 500 PSCs; HD: 2000 PSCs. Data are shown as mean ± standard error of the mean (SEM, 4–6 mice per group),*p < 0.05, **p < 0.01 and ***p < 0.001.

T1-type CD4^+^ T cells (CD4^+^IFN-γ^+^, CD4^+^TNF-α^+^) and CD8^+^ T cells (CD8^+^IFN-γ^+^, CD8^+^TNF-α^+^) were investigated in the liver (Fig. 5 and Supplementary Fig. S4). In all three groups, despite some apparent changes in T1-type CD4^+^ T cells and CD8^+^ T cells percentages with time, these changes were not significant. However, in HDG, CD4^+^IFN-γ^+^T cells percentage was significantly higher at 12 weeks than at 2 and 24 weeks (*p* = 0.0032; *p* < 0.001).

At 2 weeks, CD4^+^IFN-γ^+^T cells percentage was significantly higher in HDG than in LDG (*p* = 0.0453). At 12 weeks, CD4^+^IFN-γ^+^T cells percentage was significantly higher in HDG than in LDG and MDG (*p* < 0.001; *p* < 0.001). At 24 weeks, CD8^+^IFN-γ^+^T cells percentage was significantly higher in HDG than in LDG and MDG (*p* = 0.0045; *p* = 0.0011).

CD8^+^ TNF-α^+^T cells percentage was significantly higher in HDG than in LDG at 2 weeks (*p* = 0.0223).

### Dynamic changes of T2-type CD4 T cells in the liver of mice with different *E. granulosus s.s*. PSC inocula

T2-type CD4^+^ T cells (CD4^+^IL-4^+^) were investigated in the liver (Fig. 5 and Supplementary Fig. S4). In LDG, the percentage of T2-type cells gradually increased from the 2^nd^ to the 12^th^ week, then was lower at the 24^th^ week. In HDG, the percentage of T2-type cells was significantly higher at 12 and 24 weeks than at 2 weeks (*p* < 0.001; *p* = 0.0467).

At 2 weeks, the percentage of T2-type cells was not significantly different among all groups. At 12 weeks, the percentage of T2-type CD4^+^ T cells was significantly higher in HDG than in LDG and MDG (*p* = 0.0048; *p* < 0.001), and the percentage of T2-type cells increased with the dose of infection. At 24 weeks, the percentage of T2 cells was higher in HDG than in LDG (*p = *0.0014).

### Dynamic changes of T17-type CD4 T cells in the liver of mice with different *E. granulosus s.s*. PSC inocula

T17-type CD4^+^ T cells (CD4^+^IL-17A^+^) were investigated in the liver (Fig. 5 and Supplementary Fig. S4). In LDG,T17-type cell percentage was significantly lower at 12 weeks than at 2 weeks and 24 weeks (*p* = 0.0293; *p* = 0.0365). In HDG, T17-type cell percentage was significantly higher at 2 weeks than at 12 weeks and 24 weeks (*p* < 0.001; *p* < 0.001).

At 2 weeks, the percentage of T17-type cells was significantly higher in HDG than in LDG and MDG (p = 0.0043; p < 0.001).

### Dynamic changes of Treg-type CD4 T cells and CD8 T cells in the liver of mice with different *E. granulosus s.s*. PSC inocula

Treg-type CD4^+^ T cells (CD4^+^CD25^+^Foxp3^+^, CD4^+^IL-10^+^) and regulatory CD8^+^ T cells (CD8^+^IL-10^+^) were investigated in the liver (Fig. 5 and  Supplementary Fig. S4). In HDG, Treg-type CD4^+^ T cell percentage was significantly higher at 24 weeks than 12 weeks (*p* = 0.0016). CD8^+^IL-10^+^ T cells percentage was not significantly different in the three groups.

At 24 weeks, Treg-type CD4 T cells (CD4^+^CD25^+^Foxp3^+^) percentage was significantly higher in HDG than that in in the control group (*p* = 0.0486). At 2 weeks, CD8^+^IL-10^+^ T cells percentage was significantly higher in LDG and MDG than that in the control group (*p* = 0.0144; *p* = 0.0223).

## Discussion

Hydatid (CE) cysts, the larval stage of *E. granulosus s.s*., are able to survive in tissues, particularity in the liver of human hosts for long periods of time, often causing chronic infection^17^. The cellular distribution and phenotypes of immune mediators in the liver are distinct from those found in the periphery; analysis at the site of infection is thus key to deciphering anti-*E. granulosus s.s*. immunity. Immune responses elicited in the liver during experimental secondary infection by *E. granulosus s.l*., especially those observed at an early stage of infection, have been far less studied than those observed in *E. multilocularis* infection^12^. The aim of this study was to develop a suitable experimental model that mimics naturally infected livers to comprehensively investigate how the parasite load affects the quality of local immune response of the anti-*E. granulosus s.s*. and the outcome of the larval parasitic infection.

Consistent with previous data^18^, our study documented that mice were suitable hosts for secondary CE using PSCs obtained from sheep, and the hydatid cysts developing in the liver actually resembled those of naturally infected human liver in CE and those obtained in Balb/cJ and DBA/2 J mice by using eggs through the oral route^19^. Alternative models to peroral ingestion of eggs have been proposed in the past. Peritoneal and intravenous injections of *E. granulosus* eggs or oncospheres were proposed by Dempster *et al*. in 1991^19^: when activated oncospheres were injected intraperitoneally into Balb/cJ, DBA/2 J and CF-1 mice, cysts were restricted to the peritoneal cavity; activated oncospheres injected intravenously, however, lodged almost exclusively in the lung and thoracic cavity, except in DBA/2 J mice where 55% lodged in the liver. Different strains also exhibited different susceptibilities to secondary CE^20,21^. Our model of PSC injection has also the particular advantage of accurately mimickings secondary echinococcosis caused by local protoscolex spillage in the liver after spontaneous or therapeutic rupture of the cysts in humans. With this model, the cysts developed at a similar rate as after intraperitoneal (i.p.) inoculation with oncospheres, and macroscopic observations of the livers from our different dose groups were similar to those of the primary infection model^19^. After 4 weeks of infection, the cysts were located both at the surface of and in the liver lobes. This confirmed the initial observations made by Sahin *et al*.^22^ who established a model via the peripheral branch of the mesenteric vein; compared to that technique, the direct intraportal vein injection seems easier to perform and well reproducible. The most striking difference between the course of *E. granulosus s.s*. and *E. multilocularis* development to mature metacestodes in the same model of intra-portal injection was the absence of dose-dependent development when using *E. granulosus s.s*. PSCs. After *E. multilocularis* infection through the same route^9^, the establishment of typical *E. multilocularis* lesions was proportional to the PSC load. In fact, after *E. granulosus s.s*. PSC portal injection, macroscopic and histological examination demonstrated that at the early stage of infection (2 and 4 weeks), all infected mice, whatever the parasite load, showed minute foci in the liver caused by the parasite, thus were actually infected; however, at the middle and later stages (after 4 weeks), the foci almost disappeared and most of them healed, without significant fibrosis; only a small percentage of *E. granulosus s.s*. PSCs were able to develop into defined CE  cysts in the different dose groups, but they did develop in all groups of mice, including those receiving the lowest dose, and they were surrounded by a fibrotic AL. Such observations were previously reported in models using intraperitoneal or intravenous routes^23^. Except for the CE cysts formation, the granulomatous inflammatory responses was the most frequent pathological changes, which was in agreement with the results reported by Díaz *et al*.^24^, in which the granulomas were classified into different types. To our knowledge, this is the first time that such a course of lesions in the liver is formally described. These observations highly suggested that local cellular immunity and fibrosis were actually protective and partially able to limit or even clear PSCs in the liver; however, low antigenic stimulation was not able to trigger a totally protective host’s immune response, as it apparently did after *E. multilocularis* PSC injection, and some of the PSCs could always partially escape protective immunity and develop into cysts. Such an observation partly explains the differences in incidence of the 2 diseases in areas endemic both for AE (rarer) and CE (more frequent) despite the same definitive host (dogs) and similar levels of infectious eggs in the environment, as it happens in Western China^25^.

The terms ‘establishment’ and ‘established’ phases have been proposed to describe the developmental events occurring from the oncosphere to fully formed metacestodes and the events after metacestode formation respectively^26^. Such terms may also qualify the situation encountered in our model, in which metacestode formation comes from PSCs butnot from oncospheres. Our observations at 2 weeks fit with the definition of the ‘establishment’ phase. From the 12^th^ week the metacestodes were definitely in the ‘established phase’ in most of experimental mice. However, at the 4^th^ week, at least one metacestode (of the HDG group) was actually in the established phase, and at the 8^th^ week, several metacestodes were already in the established phase, although anumber of them were still in the establishment phase, even in the same liver, and in the same dose group, which supported  the suggestion by Mourglia-Ettlin *et al*. of a more complex parasite-host relationship than previously anticipated^26^. Evaluation of the immune cells in the establishment phase, whatever the dose-related group, showed that this phase was characterized by host’s cell granulomatous infiltration at the sites of PSCs in the liver, similar to that observed at the post-oncospheral stage in the ‘natural’ mode of infection^26^; the presence of CD19^+^ B cells, CD4^+^ T and CD8^+^ T confirmed the combined role of complement-activating anti-*Echinococcus* specific antibodies and of cellular immunity at that stage^21,26^; however, the early T2 profile, observed in all groups at 2 weeks, and especially marked in the group with high PSC load, together with a high Th17 profile in this group, as well as the early presence of CD8^+^IL-10^+^ T cells, suggested that part of CD4^+^ T and CD8^+^ T cells have a regulatory function and could play a role in the survival and growth of PSCs whatever the injection dose, especially in the high dose group; it may also be involved in the number of maturing cysts in each mouse, at later stages, which was weakly related to the parasitic load. The 4–8 week-stage thus appearedto be a crucial ‘intermediate (or transitional)’ phase which could correspond to an early or more delayed formation of the LL, susceptible to protect the developing GL of the cyst against further host’s response by tolerance induction, to allow cyst growth, and to prevent extra-cellular matrix remodelling at its contact, with the persistence of a fibrous AL around the parasitic cyst. At the established phase, the absolute numbers of NK, NKT and B cells were significantly increased in the liver of HDG mice, as was observed in previous studies after i.p. infection of mice with PSCs^26–28^, suggesting a role for NK cells in the control of mature cysts. Similar studies in CE patients hadalso shown that CD3^+^ T cells, CD3^+^CD56^+^ natural killer T cells (NKT) and B cells were the most frequent infiltrating cells at the periphery of hepatic CE  lesions; the predominance of CD3^+^-immunostained cells with weak expression of CD4^+^ and CD8^+^ subtypes in CE biopsies also highlighted the role CD3^+^CD56^+^NKTs in the partial protection towards *E. granulosus* metacestode growth by the host’s immune system; it might be involved in the differences in cyst size we observed between the LDG and the HDG^29^. The persistent presence of B cells suggested a secretion of antibodies by local B cells in the liver^20^; this could explain why specific antibodies (used for CE diagnosis) are more frequently found in patients with liver cysts than in lung cysts where the AL is less cellular^3^.

Pathological aspects of lesions in the *E. multilocularis* and in *E. granulosus s.s*. models respectively mimicked the notably different pathological aspects observed in AE and CE^3^, and, as stressed above, the outcome of *E. granulosus s.s*. infection in LDG and MDG was markedly different from that of *E. multilocularis* we reported previously, since in LDG and MDG*E. multilocularis* growth was fully cleared or considerably limited, while most of the mice of the HDG developed chronic infection^9^. In previous studies of *E. multilocularis*-infected mice, rapid inactivation of the metacestode at the very early stages, with the constitution of inflammatory and fibrotic foci morphologically similar to those observed in our *E. granulosus s.s*. model, was only observed when mice were pre-treated either with IL-12^30^ or IFN-alpha-2a^31,32^, that enhance T1-type immunity at the first stages of metacestode formation. This suggests that, despite our observations of a dominant CD8 T cell-related regulatory profile at 2 weeks, a strong stimulation of innate immunity leading to a strong T1 component of the immune response was present earlier, at the very initial stage of *E. granulosus s.s*. PSC development into metacestode, a stage that we missed in our experiments and that should be studied specifically. The absence of relationship between parasite load and the development of the AL raises complementary questions which should be addressed in further works. A definite relationship between parasite load and cytokine responses was previously shown in another experimental model of secondary CE^33^; our results with portal vein injection in C57BL/6 mice were in agreement with such previous reports, which are considered closer to the human situation in terms of resistance/susceptibility.

On comparing T cell subsets and cytokine production observed in the present study with those of *E. multilocularis* infection^9^, we could not figure out major differences in the immune cell homing and cytokine profiles between both infections; such a failure has already been stressed by reviews aimed at comparing the immune response towards both cestodes. We nevertheless observed that they exhibited some differences, summarized in Supplementary Fig. S5 for LDG, MDG and HDG respectively. At the early ‘establishment’ phase (2 weeks), the CE model presented with sequential and combined T1, T17-type T cells and Treg (CD8^+^IL-10^+^) profiles even in the LDG, while the AE model exhibited more dominant T1 and T17-type T cells subsets, associated with a nearly total parasite clearance in the LDG. In the intermediate and established phases (12 and 24 weeks), the CE model presented with a mixed T1 (CD4^+^IFN-γ^+^, CD8^+^IFN-γ^+^), T2, and T17-type T cell profile in all  different dose groups, while the AE model exhibited T1 (CD4^+^IFN-γ^+^, CD8^+^IFN-γ^+^ and CD8^+^TNF-α^+^), T2 and Treg (CD4^+^IL-10^+^, CD8^+^IL-10^+^) profiles mostly in the HDG. Among the factors that could determine both a different type of stimulation of the various types of immune response and differences at the microenvironment level, the nature of the LL in *E. granulosus s.l*. and in *E. multilocularis* respectively is a major candidate^12^. Previous studies have shown that the *E. multilocularis* LL is probably formed by a single type of mucin backbone, while a second apomucin subfamily additionally contributes to *E. granulosus s.s*. LL^34,35^. In CE, the LL seems to prevent the exogenic budding that characterizes AE lesions; in addition, as complement-activating antibodies are part of the protective immune response^36^, LL represents a first barrier against the host’s immune attack; in addition, we may hypothesize that its composition may attract and trigger the activation of the host’s cells involved in the development of the AL, which constitutes a second barrier when the cyst is fully established^12^. Different excretory/secretory metabolic products have been identified in the HF of *E. granulosus s.s*. and *E. multilocularis* respectively; recent research has shown that the presence of more enzymes in *E. granulosus s.s*. HF might be correlated with longer survival of *E. granulosus s.s*. metacestode compared to *E. multilocularis* because of an advantage in energy sources and by the protection they ensure to the parasite from the host’s immune system^37^. Progress in proteomics and in the study of differential gene expression^38^ should make the combined comparison of *E. multilocularis* and *E. granulosus s.s*. LL and HF and of the host’s immune response possible, at the very initial stages and at the single lesion level by using the quantitative intrahepatic portal vein model now well established for the two species.

## Materials and Methods

### Ethics approval and consent to participate

All mice received humane care and were used in all course of experiment according to the Animal Ethics Procedures and Guidelines of the People’s Republic of China (Regulations for Administration of Affairs Concerning Experimental Animals, China, 1988). The animal experiment was approved by The Animal Care and Use Committe and the Ethical Committe of First Affiliated Hospital of Xinjiang Medical University (20140411-05). All methods were performed in accordance with the relevant guidelines and regulations.

### Animal model and procedures

Female specific pathogen-free C57BL/6 mice of 8–10 weeks of age were obtained from the Beijing Vital River Experimental Animal Technology Co. Ltd. and were kept in the specific pathogen-free animal facility of the Research Center of the First Affiliated Hospital of Xinjiang Medical University.

*E. granulosus* *s.s.* PSCs were isolated from fresh sheep liver obtained from a local slaughterhouse, and rinsed 10 times with phosphate buffered saline, pH = 7.2, containing 1000 mg/mL penicillin and 1000 U/mL streptomycin (Hyclone,Beijing, China). Only *E. granulosus* *s.s.* PSCs inocula with a vitality over 95% confirmed by 1% methylene blue exclusion were used for portal injection in mice. The mice in experimental group were injected with different doses of PSCs via portal vein and portal saline injection was defined as control group. A middle abdominal incision was made in mice under anaesthetic and 200–300 μL of PSCs sediment or saline was injected via portal vein by using a 0.45 × 15RWLB venous infusion needle. After injection, a cotton bud was pressed on the puncture site for 5 min to provide haemostasis and to prevent intraperitoneal spillage of the PSCs. The  abdominal cavity was then closed and then the  mice were put on the warm stage to promote waking.

### Definition

To determine whether the infection percentage (or the cyst numbers) were directly related to infection dose, the mice were divided into low dose group (LDG, 50 PSCs and 250 PSCs injected through the portal vein), medium dose group (MDG, 500 PSCs), and high dose group (HDG, 1000 PSCs and 2000 PSCs).

4–6 mice in per group were sacrificed at 2, 4, 8, 12, 16, 20 and 24 weeks. Based on histological aspects and our previous research on *E. multilocularis*^9^, we selected the groups inoculated with 50 (LDG), 500 (MDG) and 2000 (HDG) PSCs to study their immune response at 2 weeks (early stage), 12 weeks (middle stage) and 24 weeks (later stage) after infection, as to further explore the correlation between inoculum doses, development of parasite-induced immune response, and resistance to infection.

The mean number (No.) cysts was defined as the total number of cysts divided by the number of mice. The infection percentage was defined as the number of infected mice with cysts, divided by number of mice in the group, multiplied by 100^19^. The formation ratio of the cysts was defined as the total number of cysts divided by the number of PSCs and the number of mice per group [total number of cysts/(number of PSCs × number of mice)], according to the previous reports^33^.

### Tissue sampling and histopathological analysis

Sixmouse hepatic lobes were separated and put in the 4% buffered formalin for 2 days, then dehydrated overnight, and finally embedded in paraffin. Paraffin-embedded 4 μm sectionson glass slides were  stained with Hematoxylin-Eosin to study inflammatory cells infiltration and granuloma and hepatic cyst formation. Histological reactions were defined as four types: (1) inflammatory foci, parasite-free, except for possible PSC remnants, composed of macrophages, lymphocytes, and other inflammatory cells; (2) fibrotic foci, parasite-free, with no visible PSCs or cysts, only composed of fibrosis, without cell infiltrates; (3) inflammatory foci with fibrosis, parasite-free, with no visible PSCs or cysts, only composed of granulomatous inflammation combined with liver fibrosis; (4) infectious foci, cystic structure composed of the GL and LL, surrounded with macrophages, lymphocytes, fibroblasts, myofibroblasts, as well as fibrosis (AL). The various parameters were  measured, expressed, and analysed as previously described^9^.

For liver fibrosis detection, paraffin-embedded 5 μm sections were stained with picric acid-Sirius red and anti-alpha smooth muscle actin antibody to respectively evaluate collagen fibers and myofibroblasts, as described previously^39^.

### Liver mononuclear cell isolation and flow cytometry analysis

Leukocytes were isolated from the liver in the way as described previously^40^. For flow cytometry, cells were stained for surface antigens with anti-NK1.1, anti-CD3, anti-CD4, anti-CD8α, anti-CD19, anti-CD44, anti-CD62L (BioLegend, San Diego, CA), or/and for intracellular antigens with anti-IFN-γ, anti-IL-4, anti-IL-17A, anti-TNF-α and anti-IL-10 (BioLegend). IgG isotype controls (Biolegend) were used in parallel. To detect Treg cells, cells were stained with anti-NK1.1, anti-CD3, anti-CD4, anti-CD25 at 4 °C for 30 minutes; and incubated with anti-mouse Foxp3 antibodies according to the manufacturer’s instructions (eBioscience). Methods were the sameas those described previously^9^.

### Statistical analysis

Data were expressed as mean ± standard error of the mean and analyzed by GraphPad Prism 7.0 (GraphPad Software, San Diego, CA). The one-way ANOVA test with a Tukey’s multiple comparison was used to test comparisons among multiple groups and the Student’s t -test was used to test comparisons between two groups, after checking for the normality of the data. If the individual values of the population were not a normal distribution and (or) unequal variances, non-parametric tests (Kruskal-Wallis test) wereused to test comparisons among groups. Correlation analysis (linear correlation) was used to test correlation between two variables. The *p*-value less than 0.05 was defined as a significant difference in this study (*p*-values were expressed in the figures as follows: *p values < 0.05; **p values < 0.01; ***p values < 0.001). Onlythose results which were statistically significant were reported in the Results section. Allresults are shown in the tables and figures.

### Ethics approval and consent to participate

All mice received humane care in all course of experiment. The animal experiment was approved by The Animal Care and Use Committe and the Ethical Committe of First Affiliated Hospital of Xinjiang Medical University (20140411-05).

## Supplementary information

Supplementary Material

## Data Availability

The datasets generated during and/or analysed during the current study are available from the corresponding author on reasonable request.
