# Supplementary material for: The local immune response during Echinococcus granulosus growth in a quantitative hepatic experimental model
Source: Sci Rep. 2019 Dec 23;9:19612. doi: 10.1038/s41598-019-56098-3 (PMC6928226; doi:10.1038/s41598-019-56098-3)
Supplement: Supplementary file 1 — Supplementary Material [file 41598_2019_56098_MOESM1_ESM.pdf]

The local immune response during *Echinococcus granulosus* growth in a quantitative hepatic experimental model

Zhide Li[1, 2, 3¶], Chuanshan Zhang[3, 4¶], Liang Li[5], Xiaojuan Bi[3], Liang Li [3], Shuting Yang[1, 3], Ning Zhang[1, 2], Hui Wang[1, 3], Ning Yang[1, 3], Abuduaini Abulizi[1, 2], Abudusalamu Aini[1, 2], Renyong Lin[3], Dominique A.Vuitton[6], Hao Wen[1,2,3*]

[1]State Key Laboratory of Pathogenesis, Prevention, Treatment of High Incidence Diseases in Central Asia, the First Affiliated Hospital of Xinjiang Medical University, Urumqi, Xinjiang, China.

[2]Department of Hepatic Hydatid and Hepatobiliary Surgery, Digestive and Vascular Surgery Centre, the First Affiliated Hospital of Xinjiang Medical University, Urumqi, Xinjiang, China.

[3]Xinjiang Key Laboratory of Echinococcosis, and WHO-Collaborating Center on Prevention and Care Management of Echinococcosis, Clinical Research Institute, the First Affiliated Hospital of Xinjiang Medical University, Urumqi, Xinjiang, China.

[4]Basic Medical College, Xinjiang Medical University, Urumqi, Xinjiang, China.

[5]Chronic Disease Laboratory, Institutes for Life Sciences and School of Medicine, South China University of Technology, Guangzhou, China

[6] French National Reference Center for Echinococcosis, Department of Parasitology, University Hospital, Besançon, France and University Bourgogne Franche-Comté (EA 3181), Besançon, France .

*Corresponding author

Email: Dr.wenhao@163.com (HW)

[¶]These authors contributed equally to this work.

Additional information

Supplementary Figure S1. Macroscopic views of the liver in mice during the course of infection. The white cysts in the livers represent established parasites. PSCs: protoscoleces. Yellow circles highlight cysts structures in the liver. Representative samples are presented.

Supplementary Figure S2. Hepatic fibrosis in infected mice during the course of infection. (a) Liver fibrosis was determined by picric acid-Sirius red staining (original magnification×100). The red area represents fibrillar collagen. (b) The fibrotic area of the section was quantified using the cellSens Dimension software, to determine the ratio of collagen area to total area (%). (c) Immunohistochemical staining for α-SMA for detection of activated hepatic stellate cells (HSCs). (d) The percentage of positive α-SMA-expressing cells to lesion areas was calculated at 2, 12 and 24 weeks post-infection. Bars indicate 100μm or 20μm in the 100× or in the 400× magnification images, respectively. Con: control; LD: 50 PSCs; MD: 500 PSCs; HD: 2000 PSCs. Data are shown as mean ± standard error of the mean (SEM, 4–6 mice per group), *$p < 0.05$, **$p < 0.01$ and ***$p < 0.001$.

Supplementary Figure S3. Hepatic memory T cell phenotypes in mice after 24 weeks-infection. Expression of CD44 and CD62L in hepatic

CD4$^+$ T and CD8$^+$ T cells from mice infected with different PSC inocula. Representative FACS plots are shown. LD: 50 PSCs; MD: 500 PSCs; HD: 2000 PSCs. Data are shown as mean values (4–6 mice per group).

Supplementary Figure S4. Representative FACS plots gated on hepatic T cell subsets in mice after 24 weeks-infection. (a) Intracellular staining of IFN-γ$^+$ in hepatic CD4$^+$ T cells from mice with different PSC inocula. (b) Intracellular staining of TNF-α$^+$ in hepatic CD4$^+$ T cells. (c) Intracellular staining of IFN-γ$^+$ in hepatic CD8$^+$ T cells. (d) Intracellular staining of TNF-α$^+$ in hepatic CD8$^+$ T cells. (e) Intracellular staining of Foxp3$^+$ in hepatic CD4$^+$ CD25$^+$ T cells. (f) Intracellular staining of IL-10$^+$ in hepatic CD8$^+$ T cells. (g) Intracellular staining of IL-4$^+$ in hepatic CD4$^+$ T cells. (h) Intracellular staining of IL-17A$^+$ in hepatic CD4$^+$ T cells. (i) Intracellular staining of IL-10$^+$ in hepatic CD4$^+$ T cells. Representative FACS plots are shown. LD: 50 PSCs; MD: 500 PSCs; HD: 2000 PSCs. Data are shown as mean values (4–6 mice per group).

Supplementary Figure S5. Comparison of T cell subsets profiles in the liver of mice during the course of infection. Data relating to *E. multilocularis* infection were quoted from the published paper by Zhang et al, 2017 [10]. For purpose of comparison, the course of T cell subsets in the low dose group, the medium dose group and the high dose group are shown in the figure.

Supplementary Table S1. Effect of *E. granulosus s.s.* PSCs on the number and distribution of cysts in mice.

Supplementary Table S2. Formation ratio and distribution of cysts in mice infected with *E. granulosus s.s.* protoscoleces (PSCs).

Supplementary Fig. S1 Macroscopic views of the liver in mice during the course of infection.

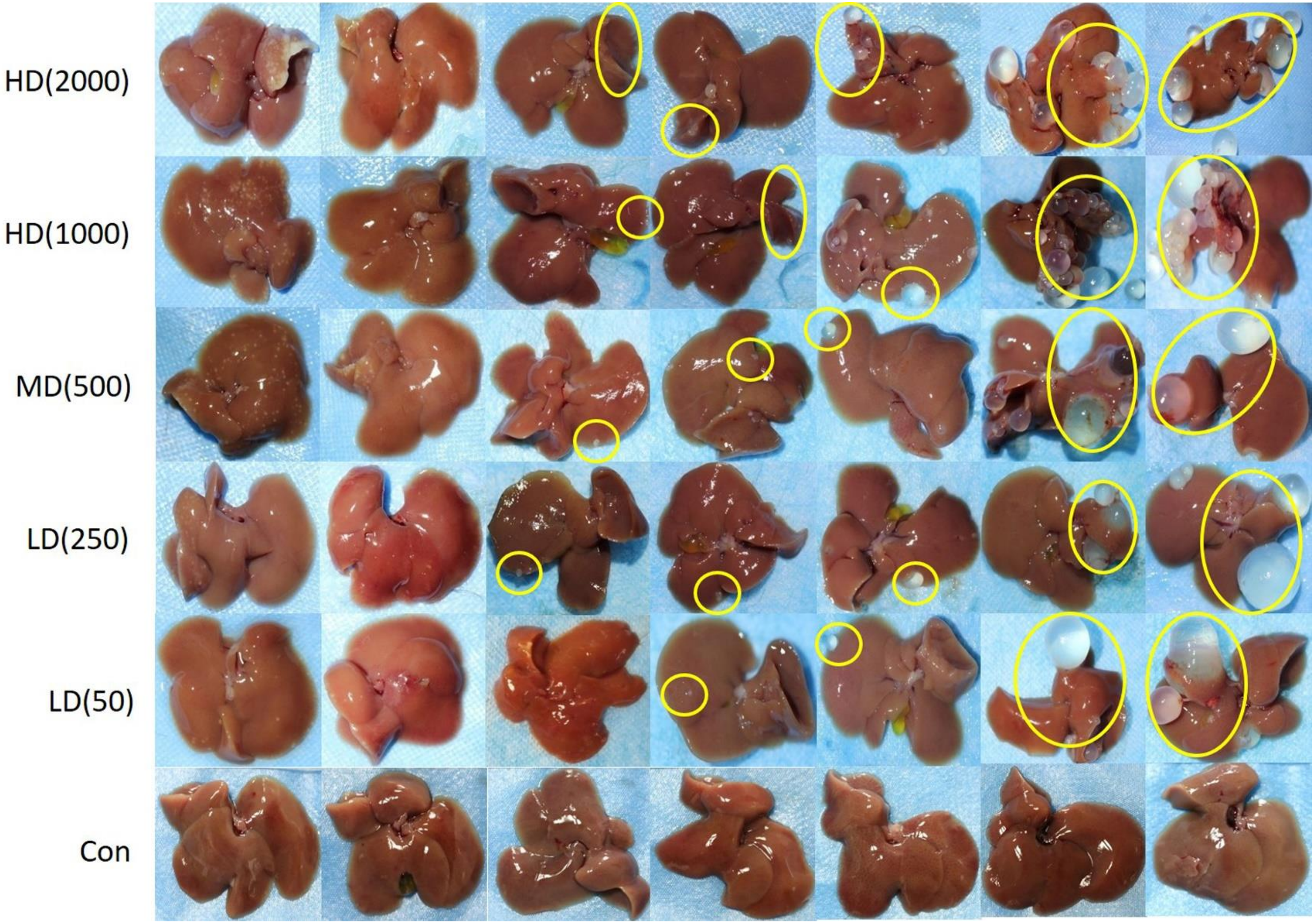

Supplementary Fig. S2 Hepatic fibrosis in mice infected during the course of infection.

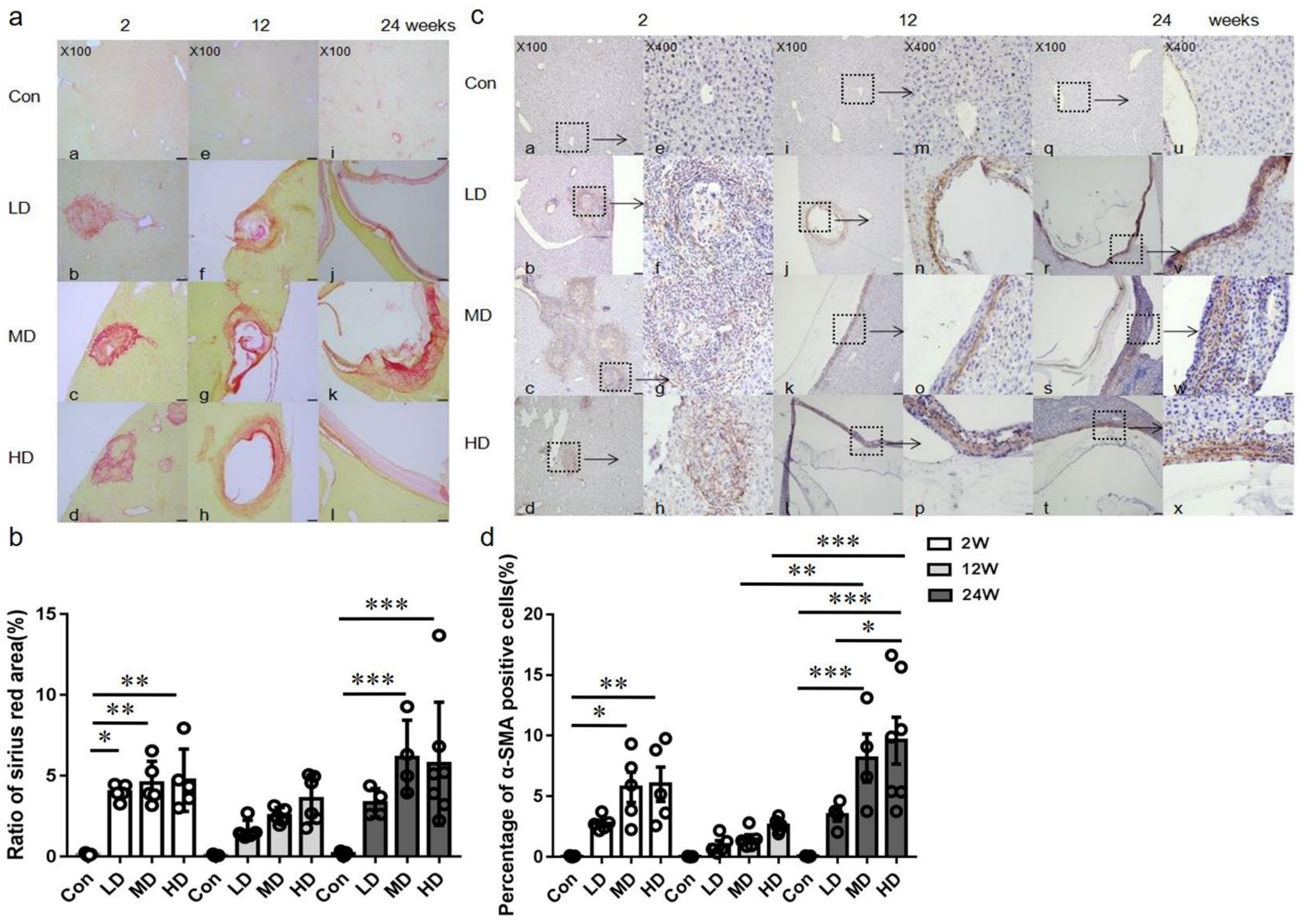

Supplementary Fig. S3 Hepatic memory T cell phenotypes in mice after 24 weeks-infection.

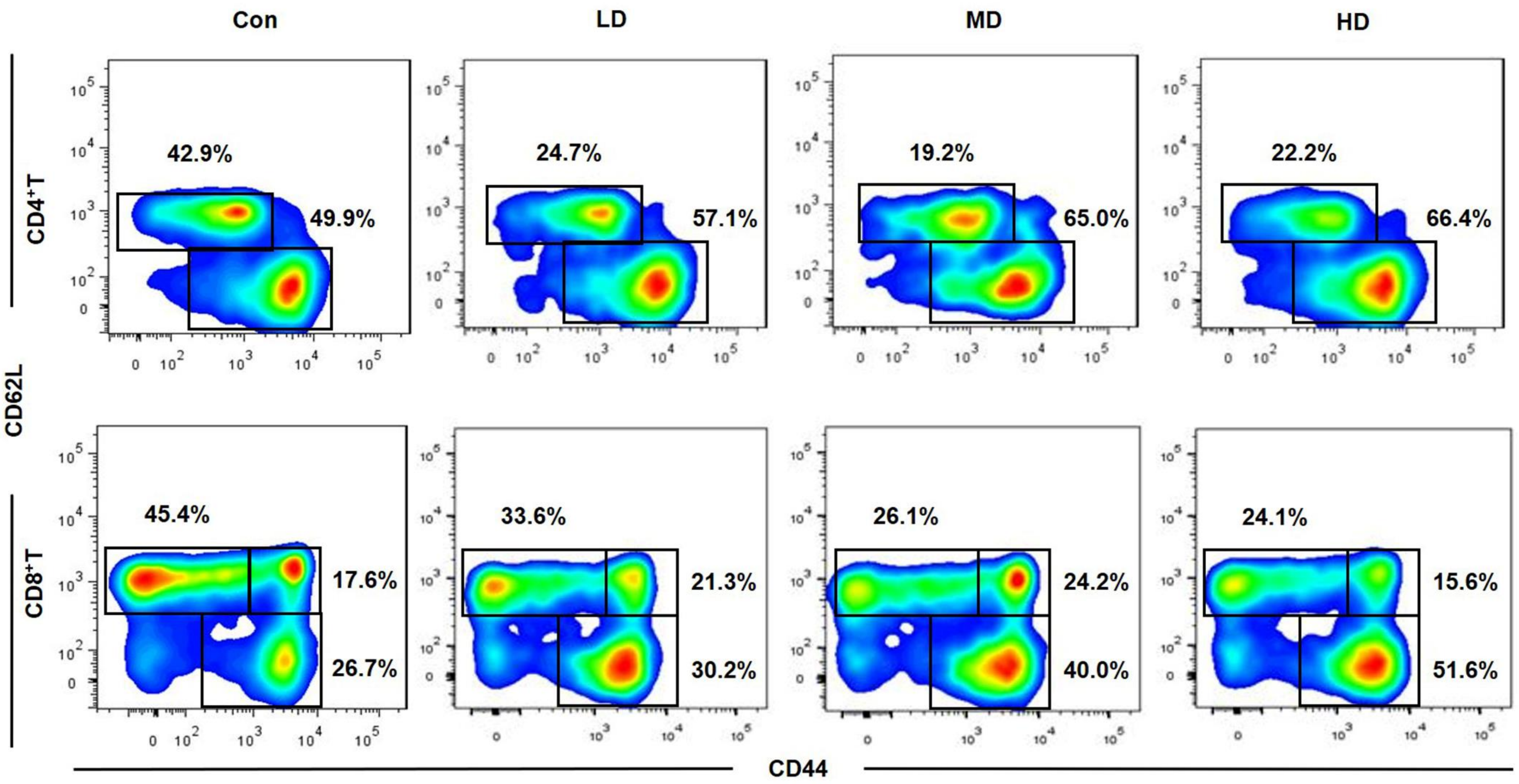

Supplementary Fig. S4 Representative FACS plots gated on hepatic T cell subsets in mice after 24 weeks-infection.

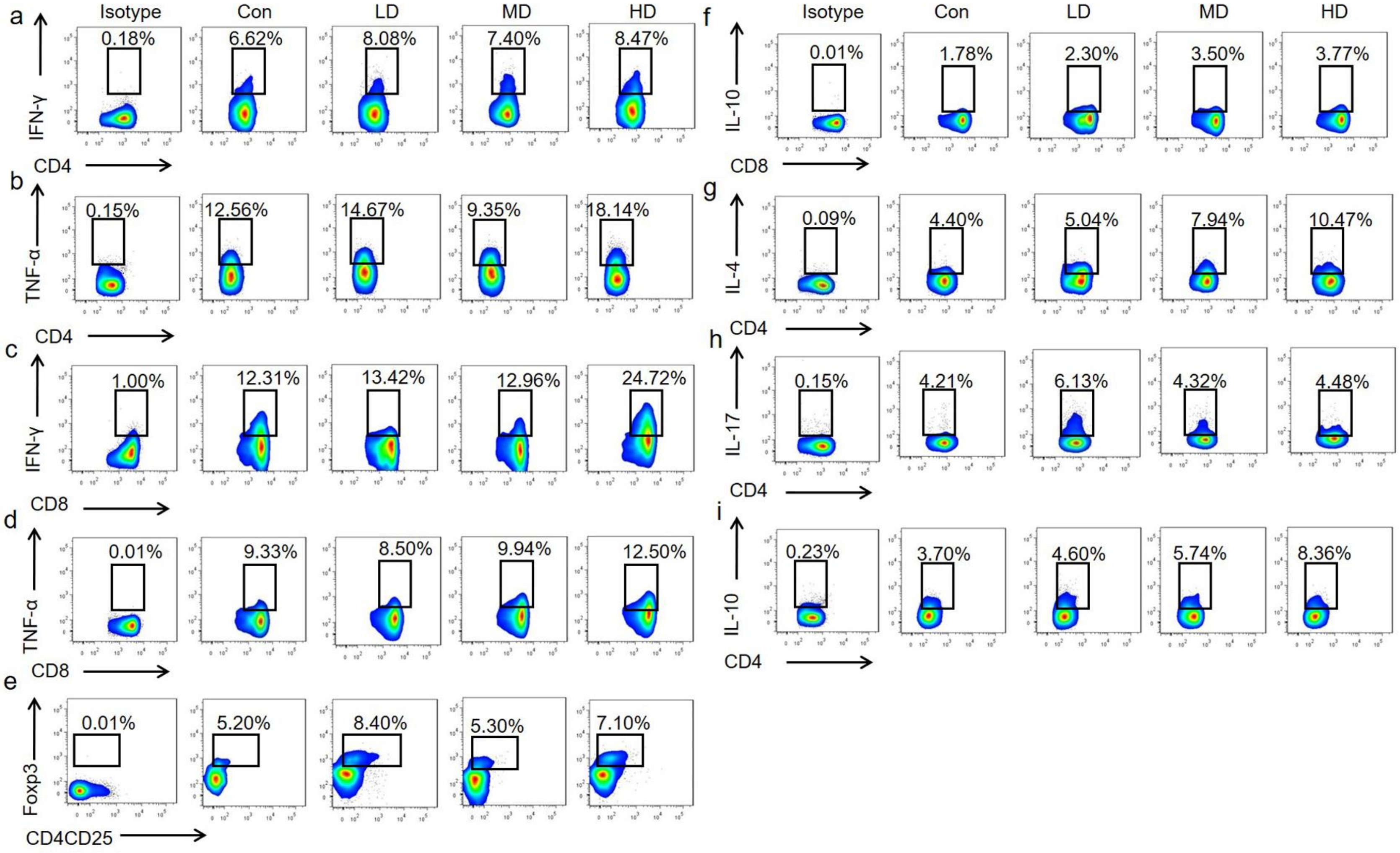

Supplementary Fig. S5 Comparison of T cell subsets profiles in the liver from mice during the course of infection.

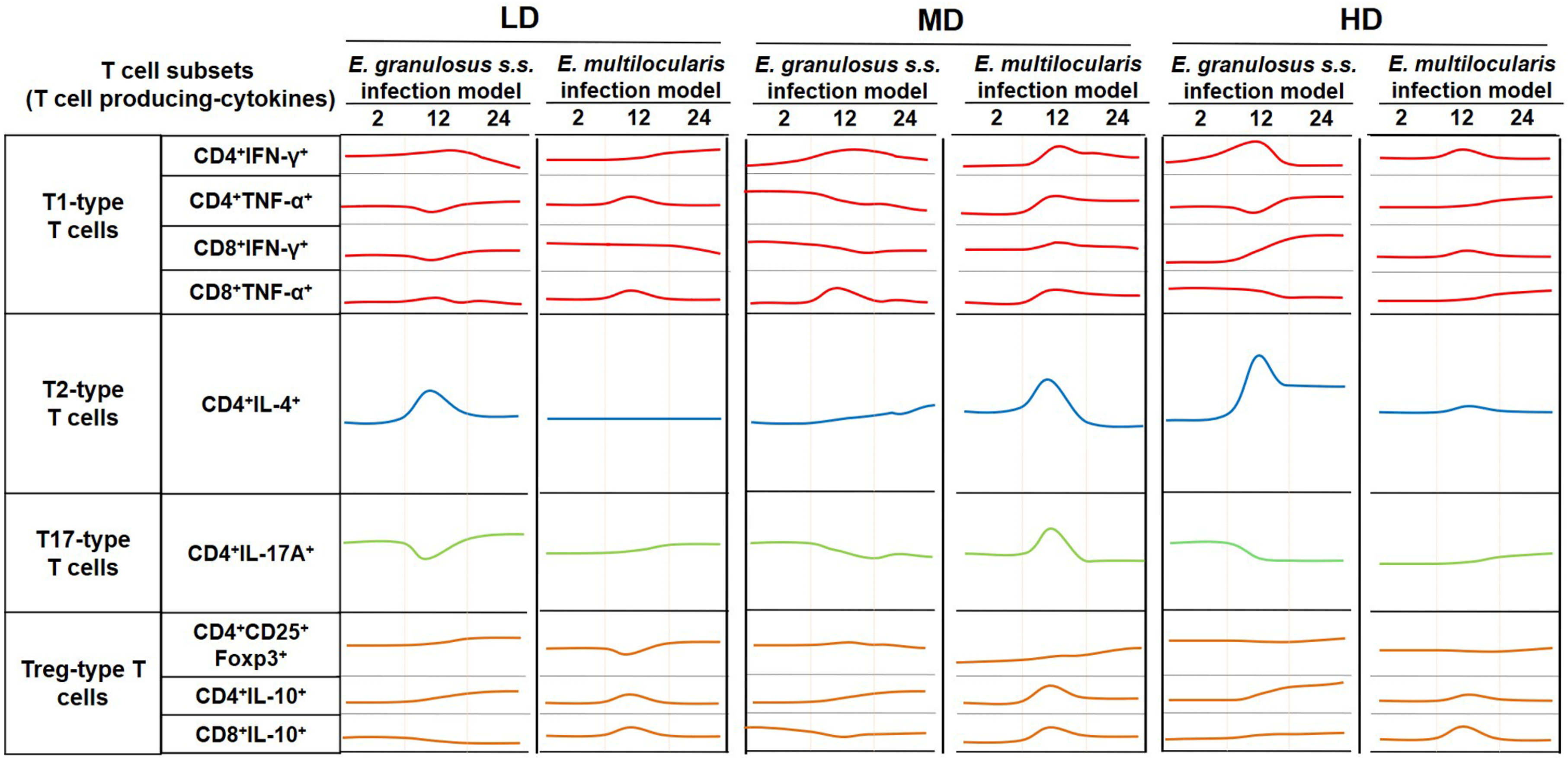

Supplementary Table S1. Effect of *E. granulosus s.s.* PSCs on the number and distribution of cysts in mice.

Time/ weeks	Group/ doses	Percentage of infection /%	No. infected mice / No. mice in group	Mean No. cysts*	Number of mice(Location/number of cysts)					
					1	2	3	4	5	6
2	50		0(0/5)	0	N	N	N	N	N	
	250		0(0/5)	0	N	N	N	N	N	
	500		0(0/5)	0	N	N	N	N	N	
	1000		0(0/5)	0	N	N	N	N	N	
	2000		0(0/5)	0	N	N	N	N	N	
4	50		0(0/5)	0	N	N	N	N	N	
	250		0(0/5)	0	N	N	N	N	N	
	500		0(0/5)	0	N	N	N	N	N	
	1000		0(0/5)	0	N	N	N	N	N	
	2000		20(1/5)	0.2	N	N	C1	N	N	
8	50		20(1/5)	0.6	N	N	M1R2	N	N	
	250		80(4/5)	0.8	L1	L1	L1	N	L1	
	500		80(4/5)	1.6	C2	N	M1C1	R1C1	R1C1	
	1000		60(3/5)	2.6	R1	L4R4C2	C2	N	N	
	2000		60(3/5)	3.0	L2C2	L6R1	N	L1C3	N	
12	50		60(3/5)	1.2	L2C1	L1	C2	N	N	
	250		100(5/5)	4.6	M1C2	L3R2C1	L2R1C7	M1R1C1	L1	
	500		100(5/5)	4.6	L2M2R3C3	L2M3C2	C1	L1	R1C3	
	1000		100(5/5)	6.6	M2R1C4	L5M5C3	L3M2C2	L1R2	L2C1	
	2000		100(5/5)	5.4	L2C5	M1R1C1	C5	C1	L1M5R2C3	
16	50		60(3/5)	2.6	N	L1	L2C3	L4R1C2	N	

	250	100(5/5)	5.4	L4M1C3	L1M2C1	M2C1	M1C4	L3R1C3	
	500	80(4/5)	7.4	N	L1C5	L5M4R5C4	M1R2C3	L1R5C1	
	1000	100(5/5)	6.6	R1C3	C1	L8M2R4C6	L3C3	C2	
	2000	80(4/5)	9.4	L1C2	L1R5C12	L4M2R1C3	N	L3M4R7C2	
20	50	100(4/4)	3.5	M2R1C3	C2	C2	C4		
	250	100(5/5)	11.2	L1C5	L4C15	L3M2C3	L3M2R6C5	C7	
	500	100(5/5)	12.6	L7R1C6	L1M1R1C1	C2	R3C13	L27	
	1000	80(4/5)	14.8	L2C10	N	L5R2C3	L1	M24C25D2	
	2000	80(4/5)	5.8	L7	L2C1	L2M5R5C4	R1C2	N	
24	50	100(4/4)	4.0	C2	C3	L2R2C4	M1C2		
	250	75(3/4)	5.75	N	L2C2D1	M3C9	M1R1C3D1		
	500	100(4/4)	14.5	C2D3	L9C11	L5M1R5C10D3	L4R3C2		
	1000	100(5/5)	16.0	L1C24D2	L2M1C3	L3M1R1C2	M13C6	R3C15D3	
	2000	100(6/6)	26.8	L2C1	D1	L1	L6M9C7	L10M9C6	L23M17R25 C42D2

*Mean No. cysts = Total number of cysts / Number of mice

N=None, L= Left lobe of liver, M = Middle lobe of liver, R = Right lobe of liver, C = Caudate lobe of liver, D = Dissociative cysts

Supplementary Table S2. Formation ratio and distribution of cysts in mice infected with *E. granulosus s.s.* PSCs.

Time/ weeks	Group/ doses	Mean ratio of cyst formation /%*	Number of cysts		
			Liver:number(percent)	Abdomen:number(percent)	Total: number
2	50	0	0(0)	0(0)	0
	250	0	0(0)	0(0)	0
	500	0	0(0)	0(0)	0
	1000	0	0(0)	0(0)	0
	2000	0	0(0)	0(0)	0
4	50	0	0(0)	0(0)	0
	250	0	0(0)	0(0)	0
	500	0	0(0)	0(0)	0
	1000	0	0(0)	0(0)	0
	2000	0.01	1(100)	0(0)	1
8	50	1.20	3(100)	0(0)	3
	250	0.32	4(100)	0(0)	4
	500	0.28	7(100)	0(0)	7
	1000	0.26	13(100)	0(0)	13
	2000	0.15	15(100)	0(0)	15
12	50	2.40	6(100)	0(0)	6
	250	1.84	23(100)	0(0)	23
	500	0.92	23(100)	0(0)	23
	1000	0.66	33(100)	0(0)	33
	2000	0.54	27(100)	0(0)	27
16	50	5.20	13(100)	0(0)	13
	250	2.16	27(100)	0(0)	27

	500	1.48	37(100)	0(0)	37
	1000	0.66	33(100)	0(0)	33
	2000	0.47	47(100)	0(0)	47
20	50	7.00	14(100)	0(0)	14
	250	4.48	56(100)	0(0)	56
	500	2.52	63(100)	0(0)	63
	1000	1.44	72(97.3)	2(2.7)	74
	2000	0.29	29(100)	0(0)	29
24	50	8.00	16(100)	0(0)	16
	250	2.10	21(91.3)	2(8.70)	23
	500	2.60	52(89.70)	6(10.30)	58
	1000	1.50	75(93.75)	5(6.25)	80
	2000	1.58	158(98.10)	3(1.90)	161

∗ Mean ratio of cyst formation (%) = Total number of cysts/ (Number of PSCs × Number of mice)
